# Angiopoietin-2 Combined with Radiochemotherapy Impedes Glioblastoma Recurrence by Acting in an Autocrine and Paracrine Manner: A Preclinical Study

**DOI:** 10.3390/cancers12123585

**Published:** 2020-11-30

**Authors:** Charly Helaine, Aurélie E. Ferré, Marine M. Leblond, Elodie A. Pérès, Myriam Bernaudin, Samuel Valable, Edwige Petit

**Affiliations:** UNICAEN, CNRS, CEA, ISTCT/CERVOxy Group, GIP Cyceron, Normandie University, 14000 Caen, France; helaine@cyceron.fr (C.H.); ferre@cyceron.fr (A.E.F.); leblond.marine.m@gmail.com (M.M.L.); peres@cyceron.fr (E.A.P.); bernaudin@cyceron.fr (M.B.); valable@cyceron.fr (S.V.)

**Keywords:** glioblastoma, angiopoietin-2, vascularization, inflammation, microenvironment, radiotherapy, chemotherapy, radiochemotherapy

## Abstract

**Simple Summary:**

Glioblastoma (GB) is a highly aggressive brain tumor characterized by poor prognosis and high rate of recurrence in response to conventional treatments consisting of tumor resection and radiochemotherapy (RCT). The reasons for this therapeutic failure are mainly due to the complexity of GB biology and its environment. GB progression is highly dependent on its vascularization and inflammatory status. Besides, evidence showed that RCT also induces vascular change and inflammation. In GB patients, Angiopoietin-2 (Ang2), biomarker of poor prognosis is a crucial angiogenic factor also involved in inflammation. Our aim was to clarify the role of Ang2 in RCT-induced changes in the GB environment. To this end, we generated Ang2-overexpressing GL261 cells and characterized tumor progression, as well as inflammation and vascularization, in response to RCT. We showed that Ang2 delays tumor recurrence and makes a lasting improvement in animal survival when combined with conventional RCT.

**Abstract:**

(1) We wanted to assess the impact of Ang2 in RCT-induced changes in the environment of glioblastoma. (2) The effect of Ang2 overexpression in tumor cells was studied in the GL261 syngeneic immunocompetent model of GB in response to fractionated RCT. (3) We showed that RCT combined with Ang2 led to tumor clearance for the GL261-Ang2 group by acting on the tumor cells as well as on both vascular and immune compartments. (4) In vitro, Ang2 overexpression in GL261 cells exposed to RCT promoted senescence and induced robust genomic instability, leading to mitotic death. (5) Coculture experiments of GL261-Ang2 cells with RAW 264.7 cells resulted in a significant increase in macrophage migration, which was abrogated by the addition of soluble Tie2 receptor. (6) Together, these preclinical results showed that, combined with RCT, Ang2 acted in an autocrine manner by increasing GB cell senescence and in a paracrine manner by acting on the innate immune system while modulating the vascular tumor compartment. On this preclinical model, we found that an ectopic expression of Ang2 combined with RCT impedes tumor recurrence.

## 1. Introduction

Glioblastoma (GB) is the most common and aggressive primary brain tumor in adults [1,2]. Standard treatment for newly diagnosed patients includes maximal safe resection followed by radiochemotherapy (RCT). RCT consists of fractionated radiotherapy (RT) combined with concomitant and adjuvant cure of chemotherapy (CT) based on temozolomide (TMZ), known as the Stupp’s regimen [3]. Despite this intensive multimodal treatment, GB patients constantly develop tumor recurrence, and their median survival rarely exceeds 15 months [4]. The limited therapeutic benefit in GB patients can be attributed to inherent tumor cell resistance but also to the tumor microenvironment, such as its vascularization [5].

GB progression is highly dependent on its vascularization, and the intense and aberrant tumor angiogenesis correlates with poor prognosis in patients [6]. The major role of the tumor microenvironment in tumor progression and treatment efficacy has led to the development of novel therapeutic options, such as antiangiogenic therapies. Due to its central role in angiogenesis, inhibition of vascular endothelial growth factor (VEGF) with bevacizumab, a humanized monoclonal antibody, was approved for recurrent GB in 2009 and recently as first-line treatment combined with RCT for newly diagnosed GB. However, while initial results with angiogenesis inhibitors were promising, they were subsequently disappointing [7,8]. Indeed, these tumors presented transient response followed by a relapse to anti-VEGF treatment [9]. This is mainly attributable to the overexpression of alternative proangiogenic factors that contribute to an adaptive resistance, as shown for angiopoietin-2 (Ang2) in GB [10,11,12,13,14].

Ang2 belongs to the angiopoietin (Ang) family consisting of two major members—Ang1 and Ang2—which act through a common tyrosine kinase receptor Tie2 but with antagonistic activities. Ang2 was originally thought to block the stabilizing effects of Ang1 on the vasculature, thereby facilitating the angiogenic response induced by VEGF or leading to vessel regression in the absence of VEGF [15]. However, conflicting results have been reported in the literature regarding the role of Ang2 in tumor angiogenesis and growth [16]. Similar contradictory results have also been reported for GB, suggesting a pro- or antiangiogenic effect of Ang2 in tumors depending on the context [17,18].

The tumor growth-supportive role of VEGF and Ang2 is not restricted to the vascular compartment and is also involved in inflammation [19]. During inflammation, endothelial cells are critically involved in regulating vascular permeability and inflammatory cell recruitment [20]. This close relationship between angiogenesis and inflammation suggests the existence of molecules that trigger both angiogenesis and the recruitment of inflammatory cells. Evidence for the capacity of cytokines to control inflammatory cell recruitment has been previously described, notably for VEGF, which attracts bone marrow-derived circulating cells [21]. In GB, the infiltrating myeloid cells, including tumor-associated macrophages (TAMs), is the major player of the innate immune system and can represent up to 30% of the tumor mass [22]. TAMs have recently been described as potential mediators of resistance to anti-VEGF therapy in these brain tumors as the degree of TAM infiltration is inversely correlated with survival among GB patients [23,24]. Ang2, which is involved in resistance to antiangiogenic therapies in recurrent GB, might also modify the tumor immune microenvironment [25,26]. This cytokine, derived from endothelial and tumor cells, may promote macrophage and neutrophil infiltration in a paracrine manner depending on either β1-integrin or Tie2, respectively [27]. In GB, Ang2 might favor leukocyte infiltration by remodeling the tumor vasculature and the blood–brain barrier integrity [12]. In particular, Ang2 has been recognized as a mediator of the homing for a subpopulation of proangiogenic TAMs, identified as Tie2-expressing macrophages (TEMs) in human GB [25,28].

Based on the key complementary roles of Ang2 and VEGF in tumor angiogenesis, strategies targeting both angiogenesis pathways were initially introduced to overcome anti-VEGF treatment resistance. Accordingly, in a murine GB model, dual inhibition of VEGF and Ang2 decreased vessel density associated with delayed tumor growth and prolongation of animal survival [29,30]. Consistent with their inflammatory-modulating function, the survival benefit of this dual therapy was also associated with TAM recruitment as well as the reprogramming of TAMs from the protumor phenotype (M2-like) toward the antitumor (M1-like) phenotype [30]. Although this multitarget therapy delays tumor growth, it fails to achieve long-term inhibition of GL261 tumor growth. More importantly, the beneficial effect of this dual therapy may be disturbed when combined with either CT or RT [31]. Vascular changes and slowdown of tumor growth were observed only when the anti-Ang2/VEGF treatment was combined with TMZ. For RT, the best efficacy was obtained with anti-VEGF alone, whereas RT is known to increase Ang2 in the brain [32]. The discrepancy between these results might reflect the context-dependent effect of Ang2. To date, the role of Ang2 in tumor microenvironment remodeling in response to RCT treatment has not been clearly established. Because Ang2 is upregulated during GB progression, as well as in response to brain RT, we speculated that this pleiotropic cytokine might play a pivotal role in the remodeling of the inflammatory environment in response to RCT. To clarify this point, we generated Ang2-overexpressing GL261 cells and characterized tumor progression, as well as inflammation and vascularization, in response to RCT.

## 2. Results

### 2.1. Ang2 Overexpression in Glioblastoma Cells Combined with Radiochemotherapy Results in Improved Animal Survival

To recapitulate the high level of Ang2 observed in GB patients [12], we generated a glioma cell line overexpressing Ang2 (GL261-Ang2) and confirmed the efficacy of the transfection at both mRNA and protein levels (Figure S1A,B). GL261-wt and GL261-Ang2 cells exhibited a similar cell cycle distribution (Figure S1C).

**Figure 1:**
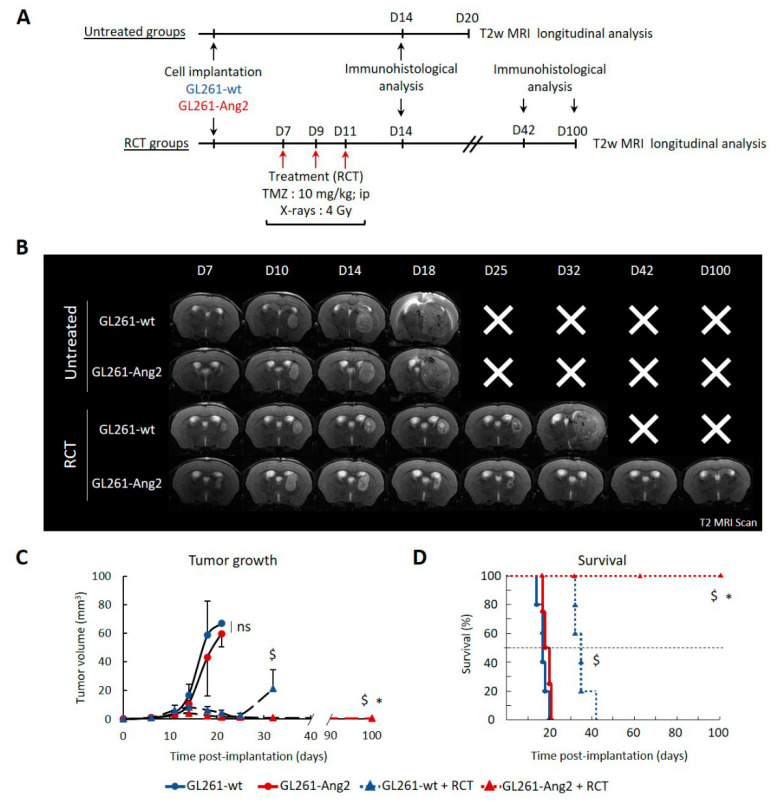
Combination of radiochemotherapy (RCT) and angiopoietin-2 (Ang2) overexpression in glioblastoma cells improved animal survival and induced complete clearance of brain tumor. (**A**) Experimental protocol to compare the tumor progression of GL261-wt and GL261-Ang2 glioblastoma models (untreated groups) and in response to RCT (RCT groups). (**B**) Representative longitudinal images in T2-weighted magnetic resonance imaging (MRI) for the four tumor groups. (**C**) Longitudinal MRI tumor volume follow-up of GL261-wt untreated (*n* = 5), GL261-wt + RCT (*n* = 5), GL261-Ang2 untreated (*n* = 4), and GL261-Ang2 + RCT (*n* = 4). Mean ± SD, * *p* < 0.05 vs. GL261-wt + RCT, $ *p* < 0.05 vs. respective untreated group, two-way ANOVA followed by Tukey’s test, ns = nonsignificant. (**D**) Comparative Kaplan–Meier survival curves for the four tumors groups: GL261-wt untreated (*n* = 5), GL261-wt + RCT (*n* = 5), GL261-Ang2 untreated (*n* = 4) and GL261-Ang2 + RCT (*n* = 4). * *p* < 0.05 vs. GL261-wt + RCT, $ *p* < 0.05 vs. respective untreated group, log-rank test.

We next studied the influence of Ang2 overexpression on tumor growth and its response to RCT following the protocol presented in Figure 1A. Tumor growth rate was monitored using T2w magnetic resonance imaging (MRI). No difference in tumor growth was observed between untreated GL261-Ang2 and GL261-wt groups (*p* = 0.11) (Figure 1B,C) while the GL261-Ang2 cell line showed a slowdown in growth rate compared to GL261-wt cells in vitro (Figure S1D).

When tumors reached an equivalent volume of about 2 mm^3^ (i.e., seven days after cell implantation), animals were treated three times with TMZ (10 mg/kg/day, i.p.) combined with RT (4 Gy/day) for 7, 9, and 11 days. Both groups showed a similar response to RCT with tumor regression 10 days after the last fraction of treatment (Figure 1C). As illustrated in MRI images (Figure 1B (D14 and D18)), the presence of necrosis in the core of tumors attested treatment-induced cell death. Although RCT led to tumor regression (*p* < 0.05), this one was transient, and tumor recurrence started 14 days after the end of treatment in the GL261-wt group. Interestingly, Ang2 overexpression potentiated the efficacy of RCT (Figure 1B and Figure S2), and this effect was maintained for at least three months post-treatment (Figure 1B,C). According to these results, there was a significant survival improvement in the treated group with the combination of Ang2 overexpression and RCT (*p* < 0.05). For the four groups of animals, the untreated GL261-Ang2 and GL261-wt had a median survival of 17 and 18 days, respectively (Figure 1D). RCT alone significantly improved the median survival to 35 days. Impressively, for Ang2 overexpression combined with RCT, the median survival was still not reached up to 100 days, and all mice were alive at this time point. At the end of the study (day 100), MRI analysis attested the complete tumor clearance (Figure 1B). We next studied whether the reduction of tumor growth was related to a vascular effect of Ang2 overexpression.

### 2.2. Ang2 Overexpression in Glioblastoma Cells Modulates the Tumor Vascular Change Induced by Radiochemotherapy

**Figure 2:**
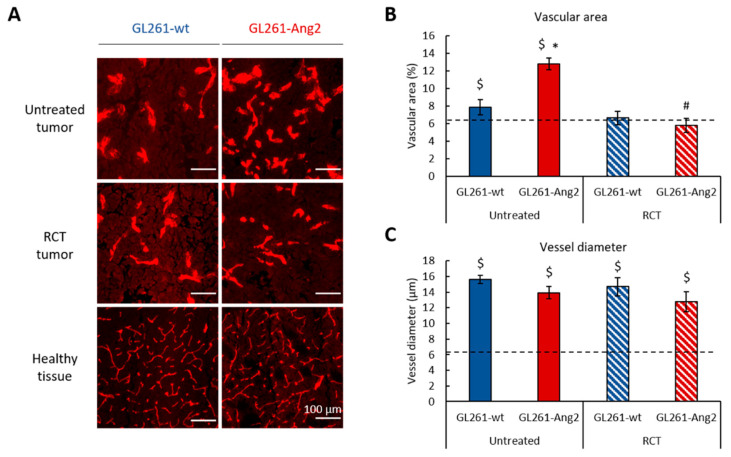
The vascularization of GL261-Ang2 tumors was more sensitive to RCT than the vascularization of GL261-wt tumors. (**A**) Representative images of CD31 immunostaining at D14 in the tumor core of GL261-wt, GL261-Ang2, and in healthy hemisphere. Scale bar = 100 µm. (**B**) Quantitative analysis of vascular area. (**C**) Quantitative analysis of vessel diameter. Dashed line corresponds to the mean of healthy tissue. *n* = 3 animals for each group. Mean ± SD * *p* < 0.05 vs. GL261-wt group, # *p* < 0.05 vs. respective untreated group, $ *p* < 0.05 vs. healthy tissue, two-way ANOVA followed by Tukey’s test.

We first examined the blood vessel morphology and density in the tumors among the four groups of animals at D14 after cell implantation (i.e., three days after the last fraction of RCT). This time was chosen because it corresponds to an early post-treatment period, allowing the detection of potential early differences in the tumor microenvironment between the groups. Figure 2A depicts the presence of typical vessels in tumors derived from the four groups and in the corresponding healthy contralateral hemisphere. Without treatment, as expected, the quantitative analysis revealed that the vascular area of GL261-wt and GL261-Ang2 tumors was larger than that of the healthy tissue (dashed line) (*p* < 0.05) (Figure 2B). These vessels were tortuous and displayed an abnormal shape, a feature of tumor vessels (Figure 2A). The quantitative analysis confirmed a larger diameter of tumor vessels (*p* < 0.05) compared to healthy tissue (Figure 2C). When tumors were exposed to RCT, the vascularization was differently affected in the two groups of animals. While the vascularization of the GL261-wt tumors was slightly affected by the treatment, the vascular density decreased significantly for GL261-Ang2 tumors treated with RCT (*p* < 0.05) (Figure 2A,B). In response to RCT, GL261-wt and GL261-Ang2 tumors displayed a vascular network similar to that of untreated GL261-wt. The vessels of the GL261-wt and GL261-Ang2 groups remained enlarged even after treatment relative to those of the healthy tissue (Figure 2C).

Collectively, these results suggest that the vascularization induced during the growth of GL261-Ang2 tumors was more sensitive to RCT than the one of GL261-wt tumors.

### 2.3. Ang2 Overexpression in Glioblastoma Cells Combined to Radiochemotherapy Favors Immune Cells Infiltration in Glioblastoma

**Figure 3:**
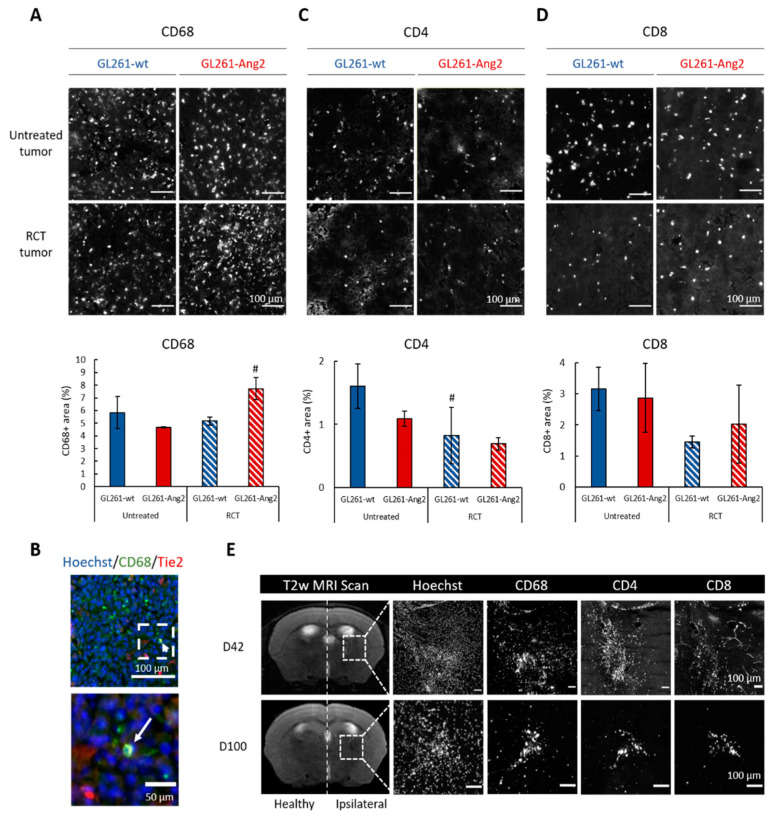
Ang2 overexpression in glioblastoma cells combined with radiochemotherapy favored inflammation in glioblastoma. (**A**) Representative images of CD68 immunostaining at D14 and quantification of CD68^+^ area. Scale bar = 100 µm. *n* = 3 animals for each group. Mean ± SD # *p* < 0.05 vs. the respective untreated group, ANOVA followed by Tukey’s test. (**B**) Representative images of CD68^+^/Tie2^+^ cells in GL261-Ang2 tumor-bearing mice. Scale bar = 100 µm and 50 µm for magnification. (**C**) Representative images of CD4 immunostaining at D14 and quantification of CD4^+^ area. Scale bar = 100 µm. *n* = 3 animals for each group. Mean ± SD # *p* < 0.05 vs. the respective untreated group, ANOVA followed by Tukey’s test. (**D**) Representative images of CD8 immunostaining at D14 and quantification of CD8^+^ area. Scale bar = 100 µm. *n* = 3 animals for each group. (**E**) Representative T2w images of the GL261-Ang2 tumor at D42 and D100 after cell implantation. Dashed lines delimit the residual tumor area and correspond to the representative images obtained from the immunohistological study to detect immune cells (CD68^+^, CD4^+^, and CD8^+^). Scale bar = 100 µm.

We then evaluated if Ang2 could affect the recruitment of immune cells into GB. A similar proportion of TAMs (CD68^+^) was identified in the two untreated groups (Figure 3A). The fractionated treatment did not change the CD68^+^ number in GL261-wt tumors (Figure 3A). In contrast, when GL261-Ang2 tumors were exposed to RCT, the TAM density increased in tumor core at D14 (7.71 ± 0.87% vs. 5.16 ± 0.32% in GL261-wt tumors, *p* < 0.05) (Figure 3A). Of note, among the CD68^+^ cells, only few were detected in the tumor cores as Tie2^+^ (Figure 3B). Fourteen days after cell implantation, no difference was observed in myeloid cell population (Table S1) in the blood and in the spleen of tumor-bearing mice (Figure S3A).

For T lymphocytes (Figure 3C,D), we did not detect any significant differences in the proportion of CD4^+^ and CD8^+^ T cells between GL261-Ang2 and GL261-wt tumors. However, a decrease of these immune cells (about 50%) was observed after RCT for both animal groups (Figure 3C,D). With or without RCT treatment, the CD4^+^/CD8^+^ ratio (around 1–1.5/3) was in favor of the CD8^+^ lymphocytes in the GL261-wt as well as in GL261-Ang2 tumors, whereas this ratio was opposite in the blood and in the spleen of tumor-bearing mice (Figure S3B and Table S1). No difference was detected in systemic inflammation irrespective of the tumor group and exposition to treatment (Figure S3A,B), even when the spleen weight of the GL261-Ang2-bearing mice was significantly higher than GL261-wt-bearing mice (*p* < 0.05) (Figure S3C).

These data suggest that, in response to RCT, GL261-Ang2 tumors display a more inflammatory environment than GL261-wt tumors. We next carried out an immunohistological study at a later time post-treatment when the tumor was no longer detectable by MRI, i.e., 42 and 100 days after cell implantation (Figure 1B and Figure 3E). As illustrated in Figure 3E, microglia/macrophages (CD68^+^) and T cells (CD4^+^ and CD8^+^) were still detected in the microscopically visible residual core of the tumor (Hoechst).

### 2.4. Ang2 Stimulates Migration of Macrophages In Vitro

**Figure 4:**
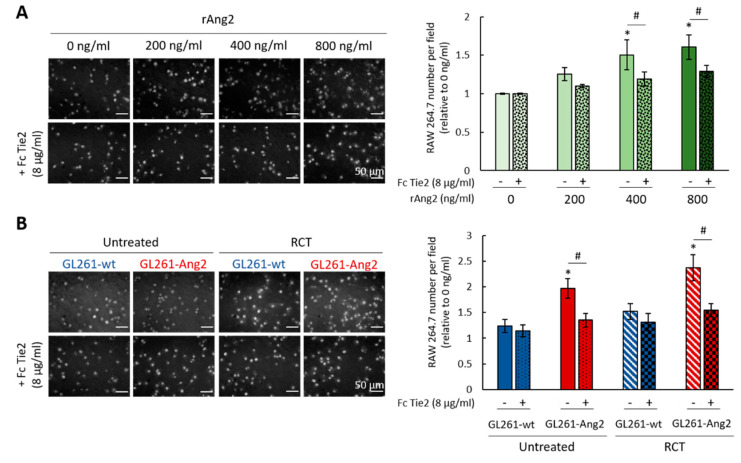
Ang2 induced macrophage migration in vitro. (**A**) Representative images and quantification of migrated RAW 264.7 cells in response to recombinant Ang2. Scale bar = 50 µm; mean ± SD, * *p* < 0.05 vs. 0 ng/mL Ang2, # *p* < 0.05 vs. Fc Tie2 condition, two-way ANOVA followed by Tukey’s test. (**B**) Representative images and quantification of migrating RAW 264.7 cells in response to conditioned medium from tumor cells. Scale bar = 50 µm; Mean ± SD, *n* = 4; * *p* < 0.05 vs. GL261-wt group, # *p* < 0.05 vs. Fc Tie2 condition, two-way ANOVA followed by Tukey’s test.

Our in vivo data suggest that Ang2 might stimulate the recruitment of myeloid cells in the murine glioblastoma model exposed to RCT. To evaluate whether Ang2 could exert a paracrine effect on macrophages, we performed in vitro migration assays with the macrophage murine RAW 264.7 cell line, known to express the two Ang2 receptors, namely, Tie2 and β1-integrin (Figure S4A,B). The migration of RAW 264.7 cells toward Ang2 was first evaluated in response to increasing concentrations of the mouse recombinant protein (rAng2) (Figure 4A). A significant effect of recombinant Ang2 appeared for concentrations greater than 400 ng/mL (*p* < 0.05). It was partially reversed by the soluble Tie2 receptor (FcTie2, *p* < 0.05) (Figure 4A), confirming the specificity of the effect of Ang2 on macrophages. We next performed similar experiments using GL261-wt or GL261-Ang2 cells exposed or not to RCT following the protocol illustrated in Figure S4C. Compared to GL261-wt, a significant increase in RAW 264.7 cell migration was detected with GL261-Ang2 cells (Figure 4B). About 1.5 times more cells migrated under these conditions (*p* < 0.05). However, this effect was similar when GL261-Ang2 cells were pre-exposed to RCT, despite a slight increase in migration of RAW 264.7 cells (Figure 4B). Importantly, like for the experiments with rAng2, the paracrine effect of Ang2 was reversed by the presence of the soluble receptor Tie2 (*p* < 0.05) (Figure 4).

These in vitro results indicate that Ang2 exerts a chemotactic effect on macrophages.

### 2.5. Ang2 Overexpression in Glioblastoma Cells Combined with Radiochemotherapy Promotes Senescence and Mitotic Death of Glioblastoma Cells

**Figure 5:**
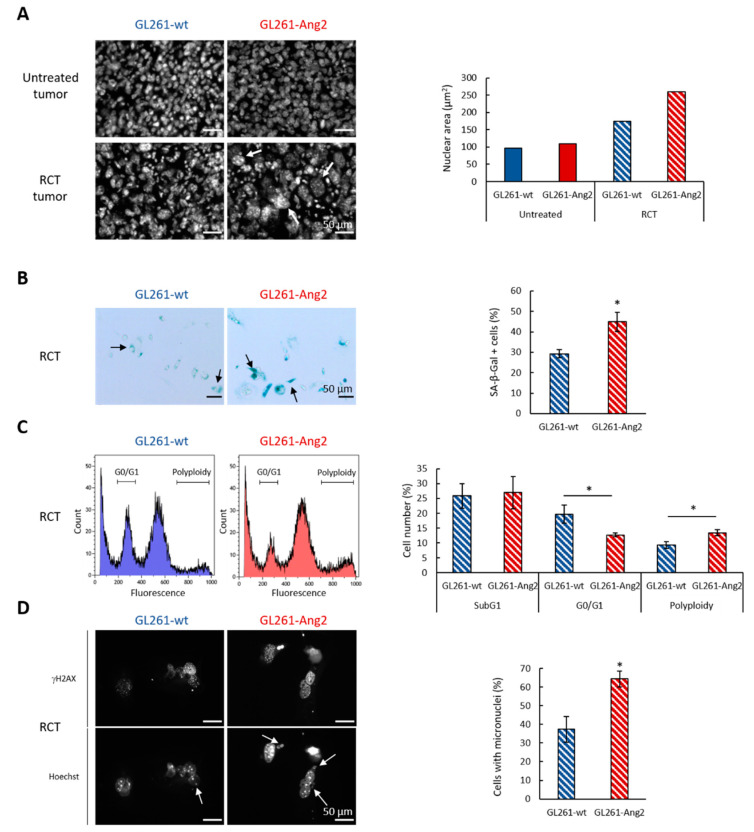
Ang2 overexpression in glioblastoma cells combined with radiochemotherapy modified cell death. (**A**) Representative photographs of nuclei (Hoechst) in the tumor core at D14 after cell implantation. Quantification of nuclei area for the four groups, *n* = 1 image/condition and the nuclei size performed on 250 nuclei/image. Scale bar = 50 µm. (**B**) Representative photographs of senescent cells detected by β-galactosidase staining performed five days following the last dose of RCT on GL261-wt and GL261-Ang2 cells. The positive cells for senescence assay showed a blue coloration. Scale bar = 50 μm. Quantification of senescence in tumor cells five days after RCT. The proportion of β-galactosidase positive cells (arrows) was expressed relative to total cell number counted by phase contrast microscopy. Mean ± SD, *n* = 3; * *p* < 0.001 vs. GL261-wt RCT group, Student’s *t*-test. (**C**) Cell cycle profile of tumor cells assessed five days after RCT. Quantification of the cell distribution in SubG1 and G0/G1 phases as well as polyploid cells five days after RCT. Mean ± SD, *n* = 3; * *p* < 0.05 vs. GL261-wt RCT group, Student’s *t*-test. (**D**) Representative photographs of DNA double-strand breaks and genomic instability linked to mitotic death and identified by the presence of micronuclei in GL261-wt and GL261-Ang2 cells five days after RCT. DNA double-strand breaks and cell micronuclei were identified with γH2AX and Hoechst staining, respectively. Scale bar = 50 μm. Quantification of mitotic death evaluated by micronuclei assay in GL261-wt and GL261-Ang2 cells five days after RCT. The proportion of positive cells with at least one micronucleus (arrows) was obtained relative to total cell number counted by Hoechst staining. Mean ± SD, *n* = 3; * *p* < 0.05 vs. GL261-wt RCT group, Student’s *t*-test.

We next investigated the nature of the sensitization to RCT induced by Ang2 overexpression in GB cells by focusing on glioma cell death. As depicted in Figure 5A, at D14 after cell implantation (i.e., three days after the last fraction of treatment), RCT induced cell nuclei changes in GL261-Ang2 as well as in GL261-wt tumors compared to the corresponding untreated tumors. These changes included enlarged nuclei with prominent foci of heterochromatin along with cellular enlargement, typical of the irradiation effects [33]. Morphometric analysis from Hoechst staining showed a two-fold increase in the nuclei size for the treated GL261-wt cells compared to untreated GL261-wt cells. However, this treatment-induced effect was particularly manifest for the GL261-Ang2 cells as the size of nuclei reached three times the one of untreated cells (Figure 5A). According to these characteristic morphological changes, we suspected a senescence phenotype for the treated tumors cells. To validate this hypothesis, GL261-wt and GL261-Ang2 cells were exposed to the identical fractionated RCT protocol that was used for in vivo experiments and were analyzed five days post-treatment. We confirmed that X-rays combined with TMZ caused the senescence of GL261-wt cells. However, this phenomenon was amplified by Ang2 overexpression as the lysosomal beta-galactosidase activity was quantitatively more significant in the GL261-Ang2 group compared to the GL261-wt group (*p* < 0.05) (Figure 5B).

Cells in senescence may return to the cell cycle, but in most cases, they may undergo cell death via apoptosis or mitotic death. Accordingly, we next addressed the question of whether Ang2 might influence these cellular processes in response to RCT. We performed a flow cytometry study for both cell lines five days post-treatment (Figure 5C). Following RCT, both tumor cells accumulated in a similar proportion in the G2/M phase (about 40% of cells) and in the subG1 phase (about 25% of cells), suggesting the presence of apoptotic cells (Figure 5C). However, at this post-RCT time, we observed a decrease of tumor cells in the G0/G1 phase, which was more marked for GL261-Ang2 (*p* < 0.05) (Figure 5C). Moreover, we noted an increase in polyploid cells in the two cell lines, but this effect was more pronounced in GL261-Ang2 cells (13.49 ± 1.09%) than in GL261-wt cells (9.28 ± 1.12%, *p* < 0.05) (Figure 5C).

To strengthen this hypothesis, we also investigated the effects of the combined treatment on DNA double-strand breaks by analyzing γH2AX positive cells. Indeed, persistence of γH2AX foci in time reflects genomic instability, which can lead to irreversible senescence (Figure 5D) [34]. One of the consequences of such genomic instability was reflected by the presence of micronuclei. As illustrated in Figure 5D, five days post-RCT, the two cell lines displayed DNA damage (γH2AX^+^ cells) and micronuclei. The cell proportion with micronuclei was higher in GL261-Ang2 cells (64.33 ± 4.16%) than in GL261-wt cells (37.33 ± 6.80%, *p* < 0.05) (Figure 5D). All together, these results suggest that Ang2 overexpression in GB cells promote mitotic cell death induced by RCT.

## 3. Discussion

In GB patients, the upregulation of Ang2 observed during the angiogenesis process and tumor progression is a biomarker of poor prognosis [12]. In these brain tumors, the angiogenic factor Ang2 has more recently been recognized as a mediator of homing for a subpopulation of macrophages identified as TEMs [25,28]. The vascular and inflammatory microenvironment of these tumors may be remodeled by the conventional treatment of GB, which includes RCT [35,36]. The main objective of our study was to evaluate whether Ang2 combined with RCT might modulate the GB microenvironment. To this end, murine GB GL261 cells were genetically modified to overexpress Ang2 and were orthotopically inoculated in mice brain. This syngeneic model allows to investigate, in addition to vascularization, the contribution of the host immune system to tumor progression [37]. In addition, the tumor biology of GL261 displays the main characteristics as that of human GB and can be used to screen the response of GL261 tumors to different therapies relevant to the treatment of GB patient, such as RCT [38].

The results obtained in this GB preclinical model were in favor of a beneficial effect of Ang2 when combined with fractionated glioma treatment combining TMZ and X-rays. In contrast, without RCT treatment, no difference was detected between tumor progression and survival of animals with GL261-Ang2 or GL261-wt tumors (Figure 1 and Figure S2). Although an autocrine Ang2 effect in tumor cells themselves was observed in vitro, as evidenced by the cell proliferation slowdown, the in vivo effects might also be attributable to its paracrine effect on the vascular and inflammatory compartments (Figure 2 and Figure 3). Indeed, we presented evidence that the overexpression of Ang2 by GB cells promoted an increase in tumor vascularization compared to control tumors in the exponential phase of growth (D14) (Figure 2). However, in the GL261-wt and GL261-Ang2 untreated tumors, the vascularization was abnormal and characterized by the presence of enlarged vessels compared to the vascularization of healthy brain tissue. At this time of tumor growth, we could not depict vascular pseudonormalization or regression that we and others had previously observed on the rat 9L gliosarcoma model using a similar in situ Ang2 overexpression approach [17,18]. Ang2 is known to have a complex and contradictory role during tumor angiogenesis. The discrepancy between these effects could be explained by the different levels of Ang2, which could lead to different outcomes [39]. However, in this GL261 model and according to Scholz et al. [12], the overexpression of Ang2 induced vascular changes but did not modify the overall survival of the mice.

In response to RCT, the vascularization tended to regress in both tumor groups to reach a similar density. This vascular regression was, however, more pronounced in the GL261-Ang2 tumor-bearing mice, and compared to untreated tumors, the decrease in the vascular area following RCT was only significant for the GL261-Ang2 group (Figure 2). In GB patients, the abnormal vascularization of tumors renders this brain tumor less susceptible to standard chemotherapy (TMZ) and radiotherapy (X-ray). Although GL261-Ang2 and GL261-wt tumors displayed disorganized and enlarged vessels, the therapeutic effect of RCT was transient in the GL261-wt tumors, whereas it was drastically prolonged in the GL261-Ang2 tumors. No recurrence was observed three months post-RCT, which corresponds to the last time point studied (Figure 1).

In addition to its function on the vasculature compartment, Ang2 also contributes to myeloid cell infiltration in settings of inflammation and tumors [12,40]. It has been previously shown that the overexpression of Ang2 in endothelial cells, which leads to an immature vascular phenotype, supports the recruitment of inflammatory cells [12]. In our model, we showed that the inoculation of GL261-Ang2 cells did not increase the presence of TAMs compared to GL261-wt tumors (Figure 3). However, in response to RCT, the TAM density specifically increased in these GL261-Ang2 tumors. The suspected chemotactic effect of Ang2 was further demonstrated in vitro in migration assays using either the recombinant cytokine or conditioned medium from GL261-Ang2 tumor cells (Figure 4). Our in vitro and in vivo results are in line with those of Cortes-Santiago et al. [25], although they also suggested that the attractant properties of Ang2 was not restricted to TEMs, as also demonstrated by Murdoch et al. [41].

The enhanced monocyte infiltration in Ang2-overexpressing tumors could be due to indirect effects via changes in the tumor-associated vasculature induced by Ang2. In the present study, Ang2 overexpression could contribute to vascular regression following RCT, which could lead to hypoxia in GL261-Ang2 tumors [40]. Hypoxia might favor monocyte extravasation, attraction, and/or retention in the tumor microenvironment in a TIE2-independent manner. Accordingly, in a previous study, we showed that the proportion of CD68^+^ cells in the tumor increased with the tumor hypoxic status [42]. Monocyte migration might also be mediated by the hypoxic upregulation of stromal cell-derived factor-1 [28], which is a potent TAM chemoattractant in various tumor models [43,44]. Nevertheless, our in vitro data from migration assay also speak in favor of direct chemoattraction of TAMs by Ang2 (Figure 4).

We confirmed that RCT led to lymphopenia but to a similar extent in both tumor groups, suggesting that Ang2 does not modify T-cell survival in these experimental conditions (Figure S3B). Others have shown that Ang2 may stimulate TEMs to acquire an immunosuppressive function in tumors by acting on Treg activation [45]. As for the vascular effect described above, we cannot exclude that the discrepancy between the results of studies on similar GL261 model might be due the different levels of Ang2 [12,40].

Interestingly, the fractionated RCT protocol used in this study in GL261-Ang2 tumors led to tumor clearance (Figure 1 and Figure S2). When we studied the cytotoxic effect of the combined therapy, we found that RCT induced glioblastoma cell senescence and that Ang2 potentiated this effect (Figure 5B). Induced senescence has been widely described for tumor cells treated with various therapeutic agents, including chemotherapies and ionizing radiation. Cells exhibiting senescence can be targeted and cleared by components of the immune system, including macrophages, NK cells, and T-lymphocytes [46]. Ang1 and Ang2 have been shown to be involved in senescence in endothelial cells [47]. However, we have shown, for the first time, that Ang2 on glioma cells might exert this effect when the cells are exposed to RCT. A role for the adaptive immune system in the elimination of senescent cells from irradiated melanoma tumors has been reported. [48]. We observed a sustained infiltration of inflammatory cells, resulting in tumor regression (Figure 3). In line with an active immune response in treated GL261-Ang2 tumors, the spleen weight of these mice indicates that these treated tumors might induce higher levels of splenocyte proliferation (Figure S3C).

RCT produced long-term survival in mice bearing GL261-Ang2 tumors. Few studies have argued in favor of a beneficial antitumor effect of this cytokine in GB models [17,18]. In contrast, Ang2 has been identified as a resistance factor to anti-VEGF [10,12,29] and also shown to mediate the homing of macrophages in human GB [12,49,50]. Recently, Ang2/VEGF bispecific antibodies were developed to overcome the anti-VEGF resistance of GB patients and stimulate the host immune as well [29,30]. In GB, immune checkpoint therapy might also benefit from Ang2/VEGF blockade. In this way, a tritherapy based on the combined inhibition of PD-1, Ang2, and VEGF might improve survival of tumor-bearing animals. This synergic therapeutic approach normalizes the tumor vessels and promotes an immunostimulatory microenvironment [51]. The divergence of the results between Di Tacchio’s study and our study might partly be due to the integration of RCT in our experimental protocol. Recently, it was proposed that conventional therapy (RT or CT) for GB patients could interfere with antiangiogenic strategies. While the combination of anti-Ang2/VEGF with TMZ chemotherapy presented beneficial effects, it was not the case for radiotherapy, which benefited only from anti-VEGF [31]. Currently, no study has evaluated the therapeutic benefit of the combination of anti-Ang2/VEGF and RCT.

The results presented here suggest that the vascular and immune properties of Ang2 probably depend not only on the concentration of this cytokine in the tumor but also on the evolution of the tumor microenvironment (vascularization and inflammatory compartment), which will be remodeled by combined radio- and chemotherapy. According to single or combined treatment (RT plus CT) and the protocols of RT (hypofractionated and normofractionated), the effects on induction or attenuation of antitumor immune response will be divergent [46,47].

Altogether, previous studies [12,45,52] and our work underline the need to include all modalities of standard treatment to better screen and characterize new therapeutics for GB.

## 4. Materials and Methods

### 4.1. Cell Culture

The murine glioblastoma cell line GL261 (NCI-DCTD (Division of Cancer Treatment and Diagnosis), Repository) and murine macrophage cell line RAW 264.7 (ATCC (American Type Culture Collections)) were used in this study. GL261 cells were maintained in RPMI medium (Roswell Park Memorial Institute, Sigma Aldrich, St Quentin Fallavier, France) supplemented with 10% fetal calf serum (Eurobio, Courtaboeuf, France), 2 mM glutamine (Gln, Sigma Aldrich, St Quentin Fallavier, France), and 1 µg/mL penicillin/streptomycin (P/S, Sigma Aldrich, St Quentin Fallavier, France) at 37 °C with 5% CO_2_ and 95% air. RAW 264.7 cells were grown in DMEM 4.5 g/L of glucose (Dulbecco’s Modified Eagle’s Medium, Sigma Aldrich, St Quentin Fallavier, France) supplemented with 10% fetal calf serum, 4 mM Gln, and 1 µg/mL P/S at 37 °C in 5% CO_2_ and 95% air.

### 4.2. Establishment of GL261 Cells Overexpressing Ang2

A pCMV6 Kan/Neo expression plasmid was employed for GL261 cells transfection. This construct vector containing murine Ang2 cDNA was transfected into GL261 cells at 80% confluence by lipofection using lipofectamine 2000 (Thermo Fisher Scientific, Illkirch-Graffenstaden, France) according to the manufacturer’s instructions. Cells expressing pCMV6-K/N-Ang2 (GL261-Ang2) were selected two days later with geneticin (G-418, Sigma Aldrich, St Quentin Fallavier, France) at 400 µg/mL. GL261-Ang2 cells were grown in RPMI medium and selected with G-418 on a monthly basis. Ang2 mRNA overexpression was routinely checked by qRT-PCR.

### 4.3. Glioblastoma Preclinical Model

Animal investigations were performed under the current European directive (2010/63/EU). Ethical approval was obtained by E.P. and S.V. from the regional committee (CENOMEXA) and the French Ministère de l’Enseignement supérieur, de la Recherche et de l’Innovation with the authorization APAFIS#12496. This tumor model is based on an orthotopic injection of GL261-wt or GL261-Ang2 cells in the right striatum of C57BL/6 mice (male, 20–23 g, Janvier Laboratories, Le Genest-Saint-Isle, France). Anesthesia in mice were induced with 5% of isoflurane in 70% N_2_O and 30% O_2_ and then maintained at 2% during surgery. Mice were placed on a stereotactic head holder, and a scalp incision was performed along the median line. A 1 mm diameter burr hole was drilled in the skull, 2 mm laterally from the bregma. Glioblastoma cells (1 × 10^5^ cells) in 3 µL of 2 mM Gln-phosphate buffer saline (PBS) were then injected at a depth of 4 mm with a 30G needle and standardized flow of 0.6 µL/min for 5 min. The needle was removed 5 min later to avoid cell reflux. Injections of buprenorphine (Buprecare, 0.05 mg/kg, sc) were administered in perioperative phases.

Tumor volumes were evaluated longitudinally by magnetic resonance imaging (7T MRI, Bruker, CYCERON biomedical imaging platform, Caen, France). Animals were anesthetized with isoflurane as described above and placed inside the magnet for acquisition with T2w sequence. The MRI scan properties were as follows: T2w rapid acquisition with relaxation enhancement (RARE), acceleration factor of 8; TR/TE_eff_ = 5000/60 msec; average = 1; 20 slices de 0.5 mm thick; spatial resolution = 0.07 × 0.07 mm; acquisition time: 2 min. MRI image analyses were completed with ImageJ software (version 1.52k) [53].

### 4.4. In Vivo Radiochemotherapy

In vivo RCT started seven days after glioblastoma cell implantation when the tumor volume was around 2–5 mm^3^. Mice were treated thrice with chemotherapy (TMZ, T2577, Sigma Aldrich, St Quentin Fallavier, France) and radiotherapy (X-rays). Each fraction of RCT was spaced at 48 h. TMZ (10 mg/kg/day in saline) was administered intraperitoneally. Control animals received the same volume of saline solution (vehicle). Two hours later, ipsilateral hemisphere of mice was irradiated with the X-RAD225 Cx system (Precision X-ray Inc, CYCERON platform, Caen, France) at 4 Gy/day with a dose rate of 3.3 Gy/min. The characteristics of the photon beam delivered by this small animal irradiator were voltage = 225 keV, intensity = 13.3 mA, and energy = 80 keV using 1 mm Cu filter. The schedule of protocol is illustrated in Figure 1A.

### 4.5. Immunohistological Analysis

At the end of the protocol, mice were deeply anesthetized and transcardially perfused with cold heparinized saline solution. Brains were removed and immediately snap-frozen in *n*-pentane (Sigma Aldrich, St Quentin Fallavier, France) and stored at −80 °C. Thereafter, brains were cut on a cryostat to obtain serial coronal sections of 30 µm. Brains sections were collected on superfrost slides (Thermo Fisher Scientific, Illkirch-Graffenstaden, France) and postfixed 20 min in 4% paraformaldehyde (PFA 4%, Sigma Aldrich, St Quentin Fallavier, France). Slices were blocked 2 h at room temperature with PBS–0.5% triton X100 (Sigma Aldrich, St Quentin Fallavier, France)–0.1% tween (Sigma Aldrich, St Quentin Fallavier, France)–3% bovine serum albumin (BSA, Sigma Aldrich, St Quentin Fallavier, France) and incubated overnight at 4 °C with the following primary antibodies: rat anti-CD31 (1:100; 553370, BD Biosciences, Le Pont-de-Claix, France), rat anti-CD68 (1:800; ab 53444, Abcam, Cambridge, UK), rat anti-CD4 (1:100; 550278, BD Biosciences, Le Pont-de-Claix, France), rabbit anti-CD8 (1:100; ab217344, Abcam, Cambridge, UK), and rabbit-anti-Tie2 (1:200; sc-324, Santa-Cruz, Heidelberg, Germany) in PBS–0.5% triton X100–0.1% tween–1% BSA. Primary antibody was detected with an Alexa-555-conjugated antirat (1:500; A21434, Invitrogen, Carlsbad, CA, USA) or an Alexa-555-conjugated antirabbit (1:500; A21428, Invitrogen, Carlsbad, CA, USA) in PBS–0.5% triton X100–0.1% tween–1% BSA containing Hoechst 33342 (10 µg/mL, Sigma Aldrich, St Quentin Fallavier, France). Slices were then coverslipped with mounting medium Aqua-PolyMount (Tebu-Bio, Le Perray-en-Yvelines, France) and acquired at ×10 magnification with epifluorescence microscope Leica DMi8S (Leitz Leica, Wetzlar, Germany).

### 4.6. Image Analysis and Quantification

All analyses were performed with regions of interest (ROI) of 736 × 736 pixels or 468 × 468 µm, chosen randomly and representative of the whole tumor. Images were then binarized with ImageJ software (version 1.52k) [53] after applying an automatic threshold. The results obtained were represented as the percentage of immunostaining in the ROI. The blood vessels were analyzed with an ImageJ (version 1.52k) plugging developed in-house [18]; this analysis provided the vascular density and the average diameter of vessels.

### 4.7. In Vitro Radiochemotherapy

For in vitro experiments, three fractions of RCT, spaced at 48 h, was applied. Tumor cells were exposed to 100 µM TMZ, which was brought in fresh medium followed by exposition to X-rays at 4 Gy with a dose rate of 2 Gy/min (Precision X-ray Inc, CYCERON platform, Caen, France).

### 4.8. Cell Cycle Analysis

Cell cycle was analyzed by flow cytometry (SFR ICORE, University of Caen platform, France). Propidium iodide (Beckman Coulter SAS, Villepinte, France) staining was realized according to the manufacturer’s instruction and detected with Gallios™ flow cytometer (Beckman Coulter SAS, Villepinte, France). All analyses were performed on a minimum of 10,000 events and analyzed with Kaluza^®^ flow analysis software (Beckman Coulter SAS, Villepinte, France).

### 4.9. SA-β-Galactosidase Assay

Senescence was determined based on alterations in cell morphology (enlargement and flattening) and expression of pH-dependent β-galactosidase [54]. Senescence associated-β-galactosidase staining (SA-β-gal, 9860S, Cell Signaling, Leiden, The Netherlands) was employed according to the manufacturer’s instructions five days after the last treatment with RCT.

### 4.10. Migration Assay

For transwell migration assay (5 µm, Sarsted, Nümbrecht, Germany), RAW 264.7 cells were plated in DMEM medium with 0.5% fetal calf serum to the top chambers of transwell, which was then added in a 24-well plate containing GL261 cells previously exposed (24 h before) to RCT. Thirty minutes before seeding RAW 264.7 cells, murine recombinant Ang2 (7186-AN, R&D Systems, Lille, France) was added at increasing doses (0, 200, 400, and 800 ng/mL) in a medium containing 0.5% serum. To evaluate Ang2 contribution, Fc Tie2 at 8 µg/mL (762-T2, R&D Systems, Lille, France) was added 30 min before adding the transwell into the plate. After withdrawing nonmigrating cells on the upper side of the membrane, migrating cells (bottom side of the membrane) were stained with Hoechst 33342 solution (10 µg/mL). Images were acquired on a Leica Aristoplan microscope (Leitz Leica, Wetzlar, Germany) with a 25× objective. Migration was quantified by counting the number of migrated cells in five microscope fields of view.

### 4.11. Immunocytochemistry

Glioblastoma cells were plated in 24-well plates on coverslips. Five days after RCT, cells were fixed with 4% PFA and blocked with PBS–0.1% tween–3% BSA for 1 h at room temperature. Then cells were incubated overnight at 4 °C with the primary antibody phosphohistone H2AX (ser13) (1:200; 2577S, Cell Signaling Technology, Leiden, The Netherlands) in PBS–0.1% Tween–1% BSA. Primary antibody was detected with an Alexa-555-conjugated antirabbit (1:500; A21428, Invitrogen, Carlsbad, CA, USA) in PBS–0.5% triton X100–0.1% tween–1% BSA. Cells were counterstained with Hoechst 33342 (10 µg/mL) for nuclear staining. A micronucleus assay was performed with Hoechst 33342 staining, and a cell with at least one micronucleus was considered positive. All immunocytochemical markers were observed on epifluorescence microscope Leica DMi8S (Leitz Leica, Wetzlar, Germany) with a 25× objective. For each condition, at least three coverslips were analyzed. Images from five representative high-power fields per slide were acquired.

### 4.12. Statistical Analysis

All data are presented as the mean ± standard deviation (SD). Statistical analyses were performed with Statistica^®^ (Tibco Software Inc, Palo Alto, CA, USA). The tests used and the number of experiments are detailed in each figure legend.

## 5. Conclusions

Our results obtained in a murine immunocompetent GB model suggest that an ectopic expression of Ang2 combined with radiochemotherapy impedes tumor recurrence. In these experimental conditions, Ang2 acted in an autocrine manner by increasing GB cell senescence and in a paracrine manner by acting on the innate immune system while modulating the vascular tumor compartment.

## Supplementary Materials

The following are available online at https://www.mdpi.com/2072-6694/12/12/3585/s1, Figure S1: Characterization of Ang2 overexpression in glioblastoma cells, Figure S2: Radiochemotherapy is more effective for glioblastoma derived from GL261-Ang2 cells, Figure S3: Involvement of Ang2 in systemic inflammation after radiochemotherapy in glioblastoma bearing mice, Figure S4: Characterization of Ang2 receptors, Tie2 and β1-integrin, in RAW 264.7 cells, Table S1: List of antibodies used for flow cytometry analyses.
