# Supplementary material for: Angiopoietin-2 Combined with Radiochemotherapy Impedes Glioblastoma Recurrence by Acting in an Autocrine and Paracrine Manner: A Preclinical Study
Source: Cancers (Basel). 2020 Nov 30;12(12):3585. doi: 10.3390/cancers12123585 (PMC7760857; doi:10.3390/cancers12123585)
Supplement: Supplementary file 1 [file cancers-12-03585-s001.pdf]

## Supplementary Materials

## 1. Methods

### *1.1. Reverse Transcription and Real Time Quantitative PCR*

The cell layer was rinsed with PBS and stored at −80 °C. The total RNA was isolated by the Nucleospin® RNA plus (Macherey-Nagel, Dueren, Germany) kit according to manufacturer's instructions. Total RNA (1 µg) were converted into cDNA during one hour at 42 °C with AMV® reverse transcriptase (Promega Corp, Madison, WI, USA). cDNA was amplified and analysed by real-time PCR with mix containing Takyon™ low ROX SYBR Mastermix (Eurogentec, Seraing, Belgium), forward and reverse mouse primers (Eurogentec): Ang1 F: 5′-CATTCTTCGCTGCCATTCTG-3′ and R: 5′-TTATATCTTCTCCCTCCGTTTTCTG-3′; Ang2 F: 5′-TTAGCACAAAGGATTCGGACAAT-3′ and R: 5′-GGACCACATGCGTCAAACC-3′; Tie2 F: 5′-GCCGCGGACTGACTACGAGC-3′ and R: 5′-GGAGGAGGGAGTCCGATAGACGC-3′; β1-integrin F: 5′-AGTGCTCCCACTTCAATCTCACCA-3′ and R: 5′-TCTCCTTGCAATGGGTCACAGGAT-3′; VEGF-A F: 5′-GGAGATCCTTCGAGGAGCACTT-3′ and R: 5′-TGGCGATTTAGCAGCAGATATAAG-3′; CD68 F: 5′-GCTTATAGCCCAAGGAACAGAG-3' and R: 5′-CTGTAGGTGTCATCGTGAAGGA-3'; Cyclophilin F: 5′-CAGACGCCACTGTGGCTTT-3′ and R: 5′-TGTGTTTGGAACTTTGTCT-3′. Amplification profile used is as follow: activation stage 3 min at 95 °C, 40 cycles of 3 sec at 95 °C and a last stage of 30 sec at 60 °C. Samples were run in triplicate and mRNA expression was calculated by the ΔCT method with QuantStudio™ 3 (Thermo Fisher Scientific, Illkirch-Graffenstaden, France).

### *1.2. ELISA Assay*

To evaluate the overexpression of Ang2 on protein level, subconfluent cells were washed in phosphate buffer saline and incubated for 48 h with 250 µL of RPMI medium. The concentration of Ang2 in the cell supernatant was measured with a mouse/rat Ang2 enzyme-linked immunosorbent assay (ELISA) kit (MANG20, R&D Systems, Lille, France), according to the manufacturer's instructions, and the optical density was measured using a Sparck plate reader (TECAN, Mannedorf, Switzerland).

### *1.3. Cell Counting*

Cells were mixed with identical volume of 0.4 % trypan blue, and the mix were counted with the hemocytometer in triplicate according to the standard methodology.

### *1.4. Immunocytofluorescence*

Immunocytofluorescence procedure is identical that previously described in materials and methods. Antibodies used for immunocytofluorescence: rat anti-CD68 (1:800; ab53444, Abcam, Cambridge, UK), rat anti-β1-integrin (1:200; MAB1997, Millipore, Molsheim, France), rabbit anti-Tie2 (1:200; sc-324, Santa-Cruz, Heidelberg, Germany).

### *1.5. Leukocyte Isolation for Flow Cytometry Analysis*

#### 1.5.1. From Blood

Blood were collected by a puncture in the ventricle just before the intracardiac perfusion procedure. The blood was recovered using a heparinized syringe, then a lysis of the red blood cells with red blood cell lysis buffer (RBC, Thermo Fisher Scientific, Illkirch-Graffenstaden, France) was carried out before immunostaining of Peripheral blood mononuclear cells (PBMCs).

1.5.2. From Spleen

The spleen was mashed through a 70 µm strainer (Sarsted, Nümbrecht, Germany). The cells were then centrifuged and resuspended in RBC lysis buffer before immunostaining of splenocytes.

1.5.3. Flow Cytometry

Cells were resuspended in 50 µL of staining buffer (Thermo Fisher Scientific, Illkirch-Graffenstaden, France) and fc receptors were blocked for 15 min at 4 °C with anti-CD16/CD32 antibodies (10 µg/mL, BD Biosciences, Le Pont-de-Claix, France). Cells were then labeled for 30 min at 4 °C with primary fluorochrome-conjugated antibodies (Table S1). Cells were washed and fixed with PBS-2 % PFA and analysed with CytoFLEX S flow cytometer (Beckman Coulter SAS, Villepinte, France).

## 2. Materials

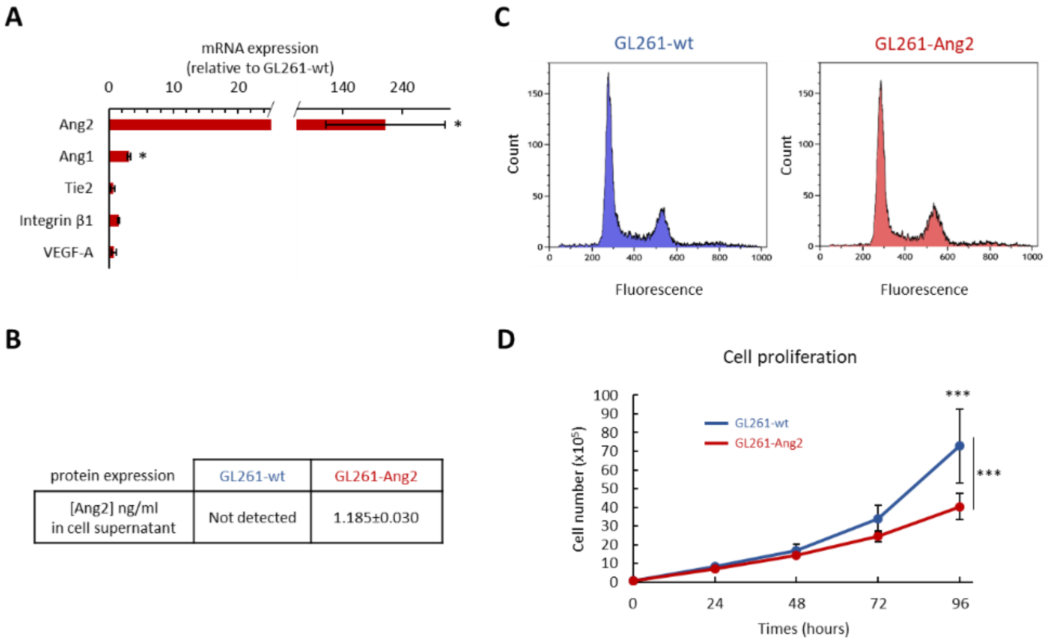


**Figure S1.** Characterization of Ang2 overexpression in glioblastoma cells. (**A**) Ang2, Ang1, Tie2, β1-integrin and VEGF-A mRNA relative expression (relative to GL261-wt) determined by qRT-PCR. Mean ± SD, N = 3, * $p < 0.05$ vs GL261-wt, Student's *t*-test; (**B**) Protein level of Ang2 secreted by tumor cells determined by ELISA assay. Mean ± SD, N = 2; (**C**) Cell cycle profile of glioma GL261-wt and GL261-Ang2 cells at 72h. (**D**) Kinetics of GL261-wt and GL261-Ang2 cells proliferation evaluated by cell counting at 24h, 48h, 72h, 96h. Mean ± SD, N = 3, *** $p < 0.001$ vs GL261-wt, ANOVA followed by Tukey's test.

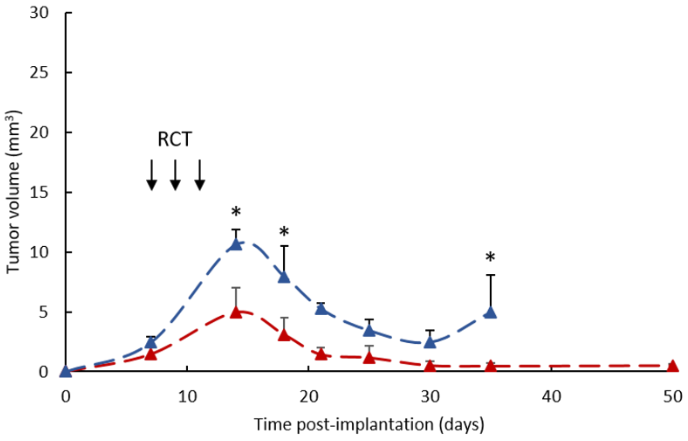


**Figure S2:** Radiochemotherapy is more effective for glioblastoma derived from GL261-Ang2 cells. Tumor volume follow-up by MRI for the two tumor groups GL261-wt + RCT (*n* = 3); GL261-Ang2 + RCT (*n* = 3); Mean ± SD, *$p < 0.05$ vs GL261-Ang2 + RCT, two-way ANOVA followed by Tukey's test.

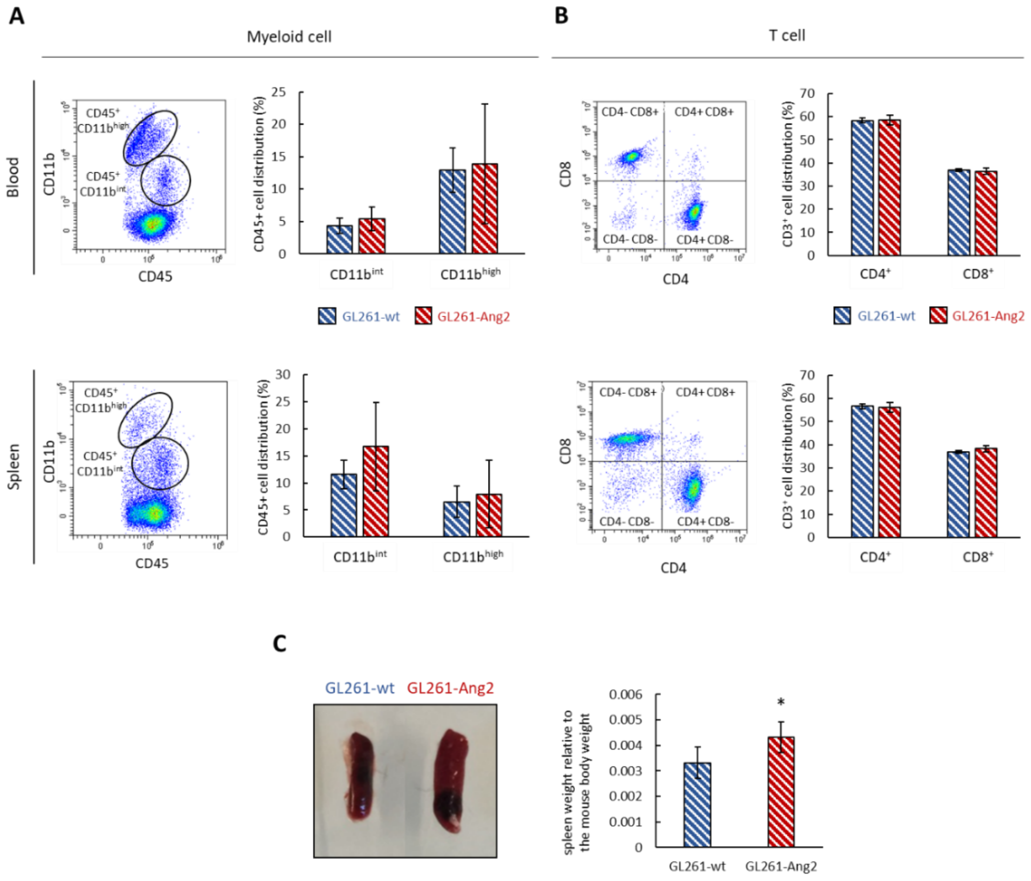


**Figure S3:** Involvement of Ang2 in systemic inflammation after radiochemotherapy in glioblastoma bearing mice. (**A**) Representative dot plots of CD11b marker in CD45$^+$ cells (myeloid cell) 14 days post-cell implantation from blood and spleen of tumor bearing mice following RCT. Mean ± SD, $n = 5$ per group, Student's *t*-test; (**B**) Representative dot plots of CD3$^+$ cells (T cell) 14 days post-cell implantation from blood and spleen of tumor bearing mice following RCT. Mean ± SD, $n = 5$ per group, Student's *t*-test; (**C**) Representative spleen's photographies of tumor bearing mice at D14. Ratio of spleen weight relative to the mouse body weight 14 days post-cell implantation of tumor bearing mice following RCT. Mean ± SD, $n = 7$ per group, * $p < 0.05$ Student's *t*-test.

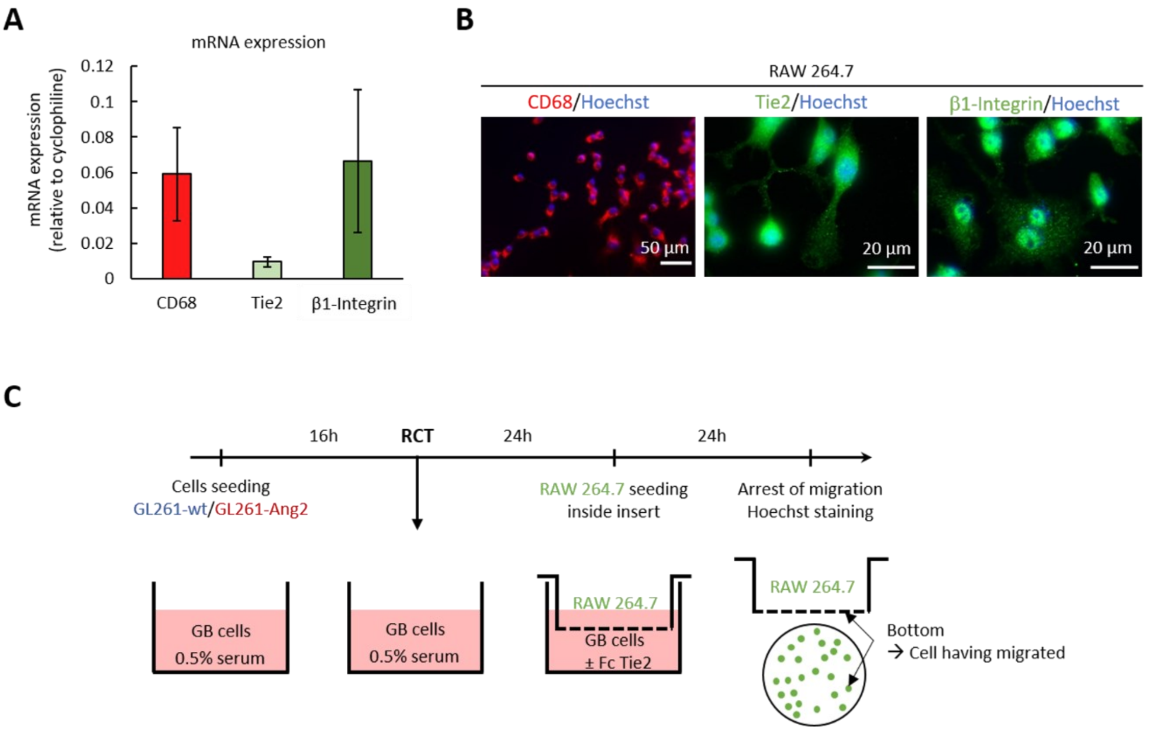


**Figure S4:** Characterization of Ang2 receptors, Tie2 and β1-integrin, in RAW 264.7 cells. (**A**) CD68, Tie2 and β1-integrin mRNA relative expressions (compared cyclophilin) in RAW 264.7 cells determined by qRT-PCR. Mean ± SD, N = 3; (**B**) Representative CD68, Tie2 and β1-integrin immunofluorescence images of RAW 264.7 cells. Scale bar for CD68 = 50 µm and scale bar for Tie2, β1-integrin = 20 µm; (**C**) Experimental protocol for RAW264.7 cells migration.

**Table S1.** List of antibodies used for flow cytometry analyses.

| Panel | | Antibody | Clone | Concentration | Supplier | Reference |
|---|---|---|---|---|---|---|
| | | CD16/CD32 | 2.4G2 | 10 µg/mL | BD Biosciences | 553142 |
| Lymphoid T cell | | V450-CD3e | 500A2 | 1 µg/mL | BD Biosciences | 560801 |
| Lymphoid T cell | | PE-CD4 | H129.19 | 1 µg/mL | BD Biosciences | 553653 |
| Lymphoid T cell | | PE-Cy™7-CD8 | 53-6.7 | 1 µg/mL | BD Biosciences | 552877 |
| | Myeloid Cell | FITC-CD45 | 30-F11 | 1 µg/mL | BD Biosciences | 553080 |
| | Myeloid Cell | PE-CD11b | M1/70 | 1 µg/mL | BD Biosciences | 553311 |
